# Risk Factors and Pathogenic Bacteria Prevalence in Catheter‐Associated Blood Stream Infection in Patients Undergoing Hemodialysis: A Cross‐Sectional Study

**DOI:** 10.1002/hsr2.72369

**Published:** 2026-04-15

**Authors:** Muhammad Shaheer Bin Faheem, Faheem Feroze, Syed Tawassul Hassan, Hafiza Qurat Ul Ain, Wajeeha Imam, Amina Saliha, Sumaya Samadi

**Affiliations:** ^1^ Karachi Institute of Medical Sciences, KIMS Karachi Sindh Pakistan; ^2^ Combined Military Hospital, CMH Rawalpindi Rawalpindi Punjab Pakistan; ^3^ Karachi Medical and Dental College, KMDC Karachi Sindh Pakistan; ^4^ CMH Multan Institute of Medical Sciences Multan Punjab Pakistan; ^5^ Kabul University of Medical Sciences “Abu Ali Ibn Sina” Kabul Afghanistan

**Keywords:** catheter‐associated blood stream infection, central venous catheter, hemodialysis, pathogenic bacteria, risk factors

## Abstract

**Background and Aims:**

Catheter‐associated bloodstream infections (CABSI) cause a significant clinical and financial burden in nephrology. This study aimed to identify the clinical risk factors and prevalent pathogens associated with CABSI among hemodialysis (HD) patients at a tertiary care hospital in Pakistan.

**Methods:**

This cross‐sectional study included 150 patients (aged 18 years or older) with CABSI undergoing HD via central venous catheters (tunneled and non‐tunneled) at CMH Hospital, Multan, over a 6‐month period. We recorded clinical details and collected dual blood samples (catheter and peripheral vein) for culture assessment. The primary outcomes were identifying clinical risk factors and determining the prevalence of causative bacterial pathogens.

**Results:**

The mean age of the sample was 61.91 ± 8.02 years, comprising 60.66% males and 39.33% females. The average duration of the catheter was 38.59 ± 18.7 days. Gram‐negative bacteria (83/150, 55.33%) were more prevalent than Gram‐positive bacteria (67/150, 44.66%) in these infections, with the most common pathogens being *Pseudomonas aeruginosa* (27/150, 18.00%) and Methicillin‐resistant *Staphylococcus aureus* (MRSA) (25/150, 16.67%). Modifiable risk factors included prolonged catheter use (> 30 days), femoral site insertion, underlying comorbidities, and anemia.

**Conclusion:**

Prolonged catheter use and femoral insertion are major modifiable risk factors for CABSI in hemodialysis patients. With Gram‐negative pathogens like *Pseudomonas aeruginosa* and MRSA predominantly responsible, targeted empirical antimicrobial strategies and early arteriovenous fistula creation are crucial to mitigate infection risks in resource‐limited settings.

## 1 Introduction

Chronic kidney disease (CKD) has a worldwide prevalence of 9.1% and is now becoming a major health concern, especially in developing nations where the cost of treatment exceeds the financial means of the majority of the affected population. Interventions, including renal replacement therapy or hemodialysis, that are needed when CKD develops to end‐stage renal disease pose a significant financial burden [1].

Hemodialysis, or a blood filtration process that maintains a stable vascular access, serves as a life‐saving treatment modality in patients with acute kidney injury (AKI) or more advanced CKD. A proper functioning of the VA is needed to achieve favorable patient outcomes after HD therapy and the overall quality of life of CKD patients. Three major types of VA for HD include the native arteriovenous fistula (AVF), the arteriovenous graft (AVG), and the central venous catheter (CVC), which is either tunneled or non‐tunneled. AVF and AVG are used for long‐term, whereas CVC is essential in cases of emergency when immediate VA is needed, and it is regarded as the necessary replacement during the continuity of the treatments in patients with HD, particularly in low‐resource settings [2, 3].

Patients on HD have a compromised immune system due to underlying medical conditions such as diabetes and malnutrition, which are worsened by uremia and dialysis procedures. Additionally, both tunnel‐cuffed and non‐cuffed catheters are associated with increased risk of infections, including exit site infections, tunnel infections, and catheter‐associated bloodstream infections (CABSI). Other severe infectious complications include endocarditis, lung abscesses, osteomyelitis, and endophthalmitis. Non‐infectious complications include central venous stenosis and catheter thrombosis [4, 5].

CABSI accounts for a significant proportion (4.2%–69%) of all infections in HD patients, leading to increased morbidity and mortality, especially in countries or settings where resources are limited [6, 7]. These infections can arise from hematogenous spread from another location, colonization of the locking solution, the skin around the insertion site, or the catheter tip [1, 8, 9]. Additional contributing factors include catheter or dialysis duration, comorbidities, site and type of catheter, inadequate hygiene, iatrogenic infections during the catheter care, and inadequate use of aseptic methods during catheter installation [10, 11]. Microorganisms such as *Staphylococcus aureus* (*S. aureus*), *Escherichia coli* (*E. coli*), *Klebsiella pneumoniae* (*K. pneumoniae*), and Coagulase‐negative staphylococci (CoNS) were highly prevalent among patients with CABSI [2, 12]. Besides these generic bacterial pathogens, candidemia has become a major catheter‐related bloodstream infection that is frequently reported among patients with CKD and associated with increased mortality in intensive care and non‐intensive care units [13, 14, 15]. Further, the use of a prolonged CVC is identified as a key precipitating factor [13, 14, 15]. These risk factors and prevalence of pathogens in CABSI are high in the low‐ and middle‐income nations and vary in different regions, different environments, and different healthcare settings [16].

Studies from Sri Lanka, Bangladesh, and Malaysia have identified these risk factors to some degree, but no such data have been determined in Pakistan, where resources in healthcare are grossly inadequate. Hence, the primary aim of this cross‐sectional research is to determine the modifiable clinical risk factors and the prevalence of causative pathogenic bacteria related to CABSI among HD patients in a tertiary care unit in Pakistan. Our findings will highlight the local epidemiological data to assist healthcare providers in optimizing VA management and adopting effective empirical antimicrobial guidelines by defining the linkage between different risk factors, catheter duration, and particular microbial infections.

## 2 Methods

### 2.1 Study Design

This cross‐sectional study included HD patients diagnosed with CABSI and was conducted at the Department of Nephrology, Combined Military Hospital, Multan, from May 1, 2024, to October 31, 2024. The study was approved by CMH Multan Ethical Review Committee (File No. 13/Trg, Ref: IEC/HD/2023/04), and informed consent was obtained from every participant.

### 2.2 Study Population

Our study comprised a total of 150 patients (aged ≥ 18 years) undergoing HD via CVC insertion and diagnosed with CABSI. Participants were selected through consecutive sampling methods, and the sample size was calculated as per the following assumptions:

Precision = 5.00%.

Prevalence of *Escherichia coli* in CABSI = 10.3%.13.

Population size = infinite, with 95% confidence interval.

Estimated sample size: *n* = 142.

Patients with ESRD, sepsis, malignancy, and AV fistula were excluded from the study. Further, those with blood cultures < 100,000 CFU and where the antibiotic use had already started before blood sample collection were also excluded.

The patient's demographic details and complete medical history were taken, and blood samples for culture were sent for pathological examinations. Blood cultures were specifically drawn when patients presented with clinical manifestations suggestive of a systemic bloodstream infection, including documented fever (≥ 38°C), unexplained chills or rigors during the hemodialysis session, or sudden hemodynamic instability. Both tunneled and non‐tunneled catheters were used and inserted by trained nephrologists via anatomical landmark technique or ultrasound. However, we did not record the detailed distribution. Routine catheter care, including connections and sterile dressing changes, was exclusively managed by the trained nephrology nursing staff at our center. Prior to accessing the catheter, strict aseptic non‐touch techniques were employed; skin preparation and catheter hub decontamination were performed using 2% chlorhexidine gluconate in 70% isopropyl alcohol/10% povidone‐iodine. Heparin was used to prime the catheters to maintain patency, and a sterile gauze dressing was used to cover the catheter exit site after the application of topical antibiotics in order to reduce the risk of infections. Furthermore, data regarding the concomitant administration of total parenteral nutrition (TPN) through the dialysis catheter were not specifically evaluated in this cohort/grounds for exclusion, as TPN was administered via separate peripheral access. A dual sampling technique was used with blood cultures obtained from both the catheter and a peripheral vein. Further catheters were removed in cases of severe CABSIs, and antibiotic therapy was initiated. A standard treatment regimen was followed, including vancomycin for Gram‐positive bacteria, while a third‐generation cephalosporin was used for the Gram‐negative bacteria. The primary outcome was determining the risk factors and the frequency of bacterial pathogens. Eligible patients were enrolled after taking written informed consent. Moreover, the patients included in our study had low hemoglobin (Hb) levels and were receiving erythropoiesis‐stimulating agent therapy along with intravenous iron supplementation in order to manage anemia.

### 2.3 Statistical Analysis

Data were analyzed using IBM SPSS Statistics, version 25.0. The normality of continuous variables (age, duration of catheter insertion, and hemoglobin levels) was assessed using the Shapiro–Wilk test, and because the continuous data were not normally distributed, they are presented as medians with interquartile ranges (IQRs), adhering to the journal's statistical guidelines. Categorical variables, such as demographic, comorbidity, and microbiological backgrounds of patients, are reported as exact frequencies and percentages. Furthermore, two‐sided Pearson Chi‐square tests were applied in order to assess the linkage of the categorical variables (catheter site, comorbidities, and gender) and the category of microbes. In addition, a multiple linear regression analysis was conducted to determine the independent effect of certain comorbidities on catheter duration. All analyses were regarded as statistically significant at a two‐sided *p*‐value below 0.05.

## 3 Results

The median age of patients in this study was 62.5 years (IQR: 57.0–67.0). The details of demographics are provided in Table 1. The median duration of catheter insertion was 30.0 days (IQR: 25.0–56.0). Clinical characteristics of patients related to HD are presented in Table 2, highlighting major clinical variables in CABSI and their association with the catheter duration. Further, the blood culture results demonstrated that the prevalence of Gram‐negative bacteria (83/150, 55.3%) was higher than that of Gram‐positive bacteria (67/150, 44.7%) among HD patients with CABSI. Moreover, *Pseudomonas aeruginosa* (27/150, 18.0%) and MRSA (25/150, 16.7%) were the most prevalent bacteria responsible for CABSI in these patients, as shown in Table 3. Multiple linear regression analysis showed that the presence of hypertension was independently associated with a significant increase in catheter duration of 7.38 days (95% CI: 1.16–13.60; *p* = 0.02). Moreover, Pearson Chi‐square analysis revealed no significant relationship between catheter insertion site and the type of microbial infection (*χ* ^2^ = 14.53, *p* = 0.56).

**TABLE 1:** Demographics.

Demographics (*n* = 150)	Value
Age	
Median (IQR) years	62.5 (57.0–67.0)
< 60 years *n*/*N* (%)	54/150 (36.0)
≥ 60 years *n*/*N* (%)	96/150 (64.0)
Gender	
Male *n*/*N* (%)	91/150 (60.7)
Female *n*/*N* (%)	59/150 (39.3)

**TABLE 2:** Clinical characteristics and catheter duration.

Clinical characteristics (*n* = 150)	Value
Co‐morbidities	
Diabetes *n*/*N* (%)	69/150 (46.0)
Hypertension *n*/*N* (%)	53/150 (35.3)
Duration of dialysis	
1 ≤ month *n*/*N* (%)	13/150 (8.7)
1–6 months *n*/*N* (%)	67/150 (44.7)
≥ 6 months *n*/*N* (%)	70/150 (46.7)
Duration of catheter insertion	
Median (IQR) days	30.0 (25.0–56.0)
1–6 days *n*/*N* (%)	12/150 (8.0)
7–30 days *n*/*N* (%)	64/150 (42.7)
> 30 days *n*/*N* (%)	74/150 (49.3)
Site of the catheter	
Femoral *n*/*N* (%)	95/150 (63.3)
Transjugular *n*/*N* (%)	51/150 (34.0)
Subclavian *n*/*N* (%)	4/150 (2.7)
Previous history of catheter‐related infections	
Yes *n*/*N* (%)	89/150 (59.3)
No *n*/*N* (%)	61/150 (40.7)
Hemoglobin	
Median (IQR) g/dL	9.0 (9.0–10.0)

**TABLE 3:** Prevalence of pathogenic bacteria.

Pathogen (*n* = 150)	Frequency *n*/*N* (%)
Gram‐negative bacteria	83/150 (55.3)
*P. aeruginosa*	27/150 (18.0)
*E. coli*	22/150 (14.7)
*A. baumannii*	20/150 (13.3)
*K. pneumoniae*	14/150 (9.3)
Gram‐positive bacteria	67/150 (44.7)
MRSA	25/150 (16.7)
MSSA	22/150 (14.7)
CoNS	12/150 (8.0)
*S. epidermidis*	6/150 (4.0)
*E. faecalis*	2/150 (1.3)

## 4 Discussion

Several studies have identified multiple risk factors for BSI in patients undergoing HD across different studies, with similar findings reported for CABSI in terms of pathogen involvement. Analysis of these studies suggests that most risk factors and pathogens are identical, and the difference depends on the region, environment, and healthcare settings/standards.

Our study aims to assess the prevalence of risk factors and pathogens associated with CABSI in HD patients, providing insights from the understudied population in Pakistan, where healthcare resources are limited. The median age of patients in our study was 62.5 years (IQR: 57.0–67.0), with males forming the majority of the included population. The median catheter duration was 30.0 days (IQR: 25.0–56.0). Our analysis revealed key epidemiological and clinical characteristics of CABSI among HD patients.

Our findings demonstrated predominant Gram‐negative bacteria (55.33%) followed by Gram‐positive bacteria (44.66%) for CABSI in HD patients. These findings are well supported by the existing literature, as Schamroth Pravda et al. showed that Gram‐negative organisms were responsible for 46.3% of bloodstream infections in HD patients, highlighting the importance of monitoring local epidemiological trends to guide effective prevention strategies [17]. Further, a study confirmed the involvement of Gram‐negative pathogens (57.6%) in the blood samples of HD patients with CABSI [2]. Conversely, a Malaysian study found a higher prevalence of Gram‐positive pathogens, primarily MRSA (17.2%), among these patients [8]. The present analysis showed that a disproportionate prevalence of CABSI (63.3%) was associated with femoral catheterization (63.3%) compared to transjugular and subclavian sites. It has been argued that this anatomical difference in infection risk is likely due to the femoral site's immediate proximity to the perineal and genitourinary tracts, which harbor rich and diverse microbial flora. Moreover, the intact and sterile dressing in the inguinal area is an inherent problem due to patient movement, skin folds in the area, and localized moisture, facilitating the quick growth of bacteria and biofilm on the extraluminal catheter surface while consequently reducing the usage of femoral catheters. However, the timely formation of arteriovenous fistulas should be prioritized for the prevention of infection. The results indicate that it is necessary to design specific empirical antimicrobial guidelines based on the local pathogen prevalence trends.

Moreover, our study revealed that comorbidities such as diabetes and hypertension significantly increase the catheter duration by 7.38 days in the presence of hypertension. This aligns with the previous findings, which suggest that diabetes contributes to the delayed or failed maturation of arteriovenous fistula, leading to increased dependence on CVCs [18, 19]. In contrast, hypertension was associated with higher AVF rates, which may be due to increased blood flow [20]. However, no significant associations were found between catheter site and pathogen type during our analysis, implying that systemic factors, including overall health and comorbidities, may surpass the anatomical site in determining infection type [2]. The association of catheter insertion site with increased risk of infection recurrence has been detailed in studies, but findings related to pathogen type remain inconsistent [8].

Our findings reflect the previous literature in terms of risk factors, showing that older age (≥ 60 years), male gender, prior catheter infections, anemia, longer HD, and catheter durations can significantly increase CABSI risk in HD patients. A study reports that males have a 2.7‐fold higher risk of developing CABSI than females, which can be attributed to the differences in vascular anatomy, hormonal effects, and health‐seeking behaviors [21]. Lafuente Cabrero et al. found that factors such as prolonged durations of catheterization, including multilumen catheters, increase the risk of CABSI, whereas single‐lumen devices lower infection rates by reducing pathogen manipulation frequency and biofilm surface area [16]. Studies have shown that CABSI was significantly associated with anemia and prior catheter infections [2, 12]. Anemia raises the infection risk by reducing oxygen supply to the tissues, impairing the immune response, while previous catheter infections compromise vascular access sites, increasing vulnerability to bloodstream infections. However, the combination of advanced age (≥ 60 years), prolonged catheter use (> 30 days), and prevalent Gram‐negative pathogens observed in our study may pre‐determine a risk profile peculiar to limited‐resources settings with delayed vascular access conversions [19]. These findings correspond to the overall variation in the HD access complications. Recently, a comprehensive 10‐year study by Arslan et al. (2025) noted a significant burden of bloodstream infections in chronic HD patients despite the usage of tunneled catheters. Their results support our data and indicate that chronic catheter dependence, irrespective of the tunneling method, is a leading and long‐lasting focus of bloodstream infections [22].

The pathogen spectrum observed in our study exhibited higher rates of Gram‐negative bacteria, primarily *P. aeruginosa* and *E. coli*, while MRSA and MSSA remained key Gram‐positive pathogens. Comparatively, some studies reported higher rates of *Acinetobacter* spp., *E. coli*, *S. aureus*, and *K. pneumoniae* in the blood samples of HD patients with CABSI [2, 12]. However, MRSA consistently remains the most prevalent Gram‐positive pathogen across different settings [8, 23, 24]. This dominance of Gram‐negative bacteria can be attributed to environmental factors, antibiotic usage patterns, and infection control practices across diverse settings, whereas the frequent occurrence of MRSA reinforces its ability to survive across diverse healthcare settings. Therefore, the choice of antibiotics during empirical treatment should follow the local antimicrobial resistance trends to ensure effective coverage against relevant Gram‐negative strains.

Our study examined a significant association of comorbidities and microbiological profiles with catheter duration and provides updated pathogen distribution trends for the South Asian HD population. These insights can inform empirical interventions, promote early AV fistula creation, and support targeting approaches to prevent infection.

## 5 Limitations

We acknowledge the study's limitations. First, the design is confined to a single center, which may introduce the risk of selection bias, as the patient population and healthcare practices, such as referral patterns, infection control, and VA management protocols, followed at our center, can vary from those in other facilities, influencing the observed risk factor and pathogen prevalence. Second, the unique geographic and socioeconomic profile of our setting may limit the generalizability of our results to high‐income settings or regions with different environments, healthcare systems, and cultural practices. Lastly, the cross‐sectional design limits our ability to establish causal relationships between risk factors and CABSI occurrence. Furthermore, while the prevalent microbial classes were identified, specific antimicrobial susceptibility and multidrug resistance profiles, particularly for the isolated Gram‐negative pathogens, were unavailable for retrospective analysis, precluding a more granular evaluation of local resistance patterns. We call for further research incorporating a multicenter longitudinal design to validate our findings and evaluate the impact of targeted therapy in reducing modifiable risk factors.

## 6 Conclusions

Long‐term CVC usage appears to be a significant modifiable risk factor for CABSI in our HD population. An early arteriovenous fistula is suggestive of reducing the reliance on temporary vascular access. There were predominant Gram‐negative bacteria as causative factors for these infections, highlighting the need for empirical interventions such as the use of antimicrobial locks to provide targeted coverage against Gram‐negative pathogens. In addition, prolonged catheter duration was associated with the presence of comorbid conditions, suggesting the integration of approaches to mitigate the risk of infection.

## Author Contributions

**Muhammad Shaheer Bin Faheem:** conceptualization, software, supervision, writing – original draft, writing – review and editing, validation, formal analysis, investigation, data curation, visualization, project administration. **Syed Tawassul Hassan:** writing – original draft, writing – review and editing, resources, validation, investigation, data curation, formal analysis. **Hafiza Qurat Ul Ain, Wajeeha Imam, Amina Saliha:** conceptualization, writing – original draft, writing – review and editing, project administration, data curation, resources, validation, methodology. **Sumaya Samadi:** resources, methodology, project administration.

## Funding

The authors have nothing to report.

## Ethics Statement

This study was conducted in accordance with the Declaration of Helsinki. Ethical approval for this retrospective analysis was obtained from the Institutional Review Board and Ethics Committee of CMH Multan (File No. 13/Trg; Ref: IEC/HD/2023/04). Given the retrospective nature of the study and the use of de‐identified patient data, the requirement for written informed consent was waived by the ethics committee.

## Consent

The authors attest that the participants were aware of the study's purpose, risks, and benefits.

## Conflicts of Interest

The authors declare no conflicts of interest.

## Transparency Statement

The lead author Sumaya Samadi affirms that this manuscript is an honest, accurate, and transparent account of the study being reported; that no important aspects of the study have been omitted; and that any discrepancies from the study as planned (and, if relevant, registered) have been explained.

## Data Availability

The data that support the findings of this study are available from the corresponding author upon reasonable request.
