# Correction to “Risk Factors and Pathogenic Bacteria Prevalence in Catheter‐Associated Blood Stream Infection in Patients Undergoing Hemodialysis: A Cross‐Sectional Study”

**DOI:** 10.1002/hsr2.72735

**Published:** 2026-06-30

M. S. B. Faheem, F. Feroze, S. T. Hassan, et al., “Risk Factors and Pathogenic Bacteria Prevalence in Catheter‐Associated Blood Stream Infection in Patients Undergoing Hemodialysis: A Cross‐Sectional Study,” *Health Science Reports* 9 (2026): e72369, https://doi.org/10.1002/hsr2.72369.

In the **Author Contributions** section, the information was incomplete and has been updated to include the contributions of the author Faheem Feroze as follows:

**Muhammad Shaheer Bin Faheem**: conceptualization, software, supervision, writing – original draft, writing – review and editing, validation, formal analysis, investigation, data curation, visualization, project administration. **Faheem Feroze and Syed Tawassul Hassan**: writing – original draft, writing – review and editing, resources, validation, investigation, data curation, formal analysis**. Hafiza Qurat Ul Ain, Wajeeha Imam and Amina Saliha:** conceptualization, writing – original draft, writing – review and editing, project administration, data curation, resources, validation, methodology. **Sumaya Samadi**: resources, methodology, project administration.

We apologize for this error.

